# Identification of miRNA-mRNA crosstalk in CD4^+^ T cells during HIV-1 infection by integrating transcriptome analyses

**DOI:** 10.1186/s12967-017-1130-y

**Published:** 2017-02-21

**Authors:** Qibin Liao, Jin Wang, Zenglin Pei, Jianqing Xu, Xiaoyan Zhang

**Affiliations:** 1 Shanghai Public Health Clinical Center, Fudan University, Shanghai, China; 2 Institutes of Biomedical Sciences, Key Laboratory of Medical Molecular Virology of Ministry of Education/Health, Fudan University, Shanghai, China

**Keywords:** HIV-1, Clinical outcome, Integrative transcriptome analyses

## Abstract

**Background:**

HIV-1-infected long-term nonprogressors (LTNPs) are characterized by infection with HIV-1 more than 7–10 years, but keeping high CD4^+^ T cell counts and low viral load in the absence of antiretroviral treatment, while loss of CD4^+^ T cells and high viral load were observed in the most of HIV-1-infected individuals with chronic progressors (CPs) However, the mechanisms of different clinical outcomes in HIV-1 infection needs to be further resolved.

**Methods:**

To identify microRNAs (miRNAs) and their target genes related to distinct clinical outcomes in HIV-1 infection, we performed the integrative transcriptome analyses in two series GSE24022 and GSE6740 by GEO2R, R, TargetScan, miRDB, and Cytoscape softwares. The functional pathways of these differentially expressed miRNAs (DEMs) targeting genes were further analyzed with DAVID.

**Results:**

We identified that 7 and 19 DEMs in CD4^+^ T cells of LTNPs and CPs, respectively, compared with uninfected controls (UCs), but only miR-630 was higher in CPs than that in LTNPs. Further, 478 and 799 differentially expressed genes (DEGs) were identified in the group of LTNPs and CPs, respectively, compared with UCs. Compared to CPs, four hundred and twenty-four DEGs were identified in LTNPs. Functional pathway analyses revealed that a close connection with miRNA-mRNA in HIV-1 infection that DEGs were involved in response to virus and immune system process, and RIG-I-like receptor signaling pathway, whose DEMs or DEGs will be novel biomarkers for prediction of clinical outcomes and therapeutic targets for HIV-1.

**Conclusions:**

Integrative transcriptome analyses showed that distinct transcriptional profiles in CD4^+^ T cells are associated with different clinical outcomes during HIV-1 infection, and we identified a circulating miR-630 with potential to predict disease progression, which is necessary to further confirm our findings in the future.

**Electronic supplementary material:**

The online version of this article (doi:10.1186/s12967-017-1130-y) contains supplementary material, which is available to authorized users.

## Background

HIV-1 infection is characterized by the loss of number and dysfunction of CD4^+^ T cells and exhibits remarkable differences in clinical outcomes of treatment-naïve individuals [1]. As chronic progressors (CPs) or normal progressors (NPs), the majority of HIV-1-infected patients with progressive virus replication have chronic loss of CD4^+^ T cells and develop to AIDS in several years without any antiretroviral therapy (ART) [2, 3]. However, long-term nonprogressors (LTNPs) (≈5% of HIV-1-infected individuals), without progression of AIDS, maintain normal counts of CD4^+^ T cells (>500 cells/μl) and low viral load (LVL) without ART for many years [4, 5]. Moreover, several studies have found that LTNPs display a higher level of HIV-specific CD4^+^ and CD8^+^ T cell responses than that in chronic progressors [6, 7], which greatly slows disease progression to AIDS [5, 8, 9]. Although there are some known protective factors involved inHIV-1 disease progression or pathogenesis, such as specific protective HLA-B*57/B*27 alleles [10], the *CCR5*delta32 [11] and defective viruses [12] in LTNPs, the mechanisms of nonprogression in HIV-1 infection remains to be further explored.

MiRNAs are a class of small non-coding RNAs with the length of ≈22 nucleotides, which plays important roles in post-transcriptional regulation of genes. MiRNAs function to pair to 3′-untranslated regions (3′-UTR) of target mRNA, and almost all of miRNAs result in decreased target mRNA levels and/or protein translated [13]. MiRNAs have been demonstrated to suppress HIV-1 via decreasing HIV dependency factors (HDFs), miR-198 targets Cyclin T1 [14], miR-17/92 regulates P300/CBP-associated factor (PCAF) [15], and miR-15a/b, miR-16, miR-20a, miR-93, miR-106b bind to Pur-α and repress its expression [16]. It has also been proposed that miRNAs could either directly bind to HIV-1 RNA or affect cellular factors involved in HIV-1 replication [17]. MiRNAs can also modulate key regulatory molecules related to T cell exhaustion following HIV-1 infection [18]. MiR-9 regulates the expression level of Blimp-1 that considered as a T cell exhaustion marker [19], and let-7 miRNAs play a regulatory role in post-transcription of an immune inhibitory molecule, IL-10 [20]. MiR-125b, miR-150, miR-223, miR-28 and miR-382 [21], and miR-29a [22] have high abundance in resting CD4^+^ T cells, which contributes to inhibition of HIV-1. Furthermore, several miRNAs in peripheral blood mononuclear cells (PBMC) and plasma can predict the disease progression of HIV-1 infection, such as miR-31, miR-200c, miR-526a, miR-99a, miR-503 [23], and miR-150 [24]. Therefore, identification of deregulated miRNA expression profiles in different clinical outcomes of HIV-1 infection may be useful for further understanding the possible mechanisms associated with disease progression, pathogenesis and immunologic control.

However, there is no evidence that miRNA-mRNA co-expression profiles in different clinical outcomes of HIV-1 infection. Considering that CD4^+^ T cells are target cells of HIV-1 and the CD4^+^ T cell counts is employed to surveiller disease progression, we integrated miRNA and transcriptomic expression profiles data of CD4^+^ T cells in two series selected from GEO datasets in order to identify miRNA-mRNA crosstalk in HIV-1 infection. We have found numerous HIV-1 disease progression and pathogenesis-associated miRNAs and differentially regulated genes, then we constructed functional network of potential miRNA-mRNA pairs. Identification of genetic and/or epigenetic biomarkers may not only facilitate understanding of interaction between HIV-1 and host CD4^+^ T cells, but lead to develop novel markers for prediction of disease progression or therapeutic targets for HIV-1.

## Methods

### Dataset collection

The series GSE6740 was downloaded from the Gene Expression Omnibus (GEO) datasets (http://www.ncbi.nlm.nih.gov/geo/), contained 15 gene chips from 5 uninfected controls (UCs), 5 chronic progressors (CPs) and 5 long-term nonprogressors (LTNPs), which was analyzed using the platform, GPL96 (HG-U133A) Affymetrix Human Genome U133A Array. The series GSE24022 included miRNA microarray data of CD4^+^ T cells from 8 UCs, 7 LTNPs and 7 CPs, whose platform is Agilent-019118 Human miRNA Microarray 2.0 G4470B (miRNA ID version). These samples in the aforementioned series were divided into three comparison groups to perform subsequent analyses: the group of LTNPs versus UCs, CPs versus UCs, and LTNPs versus CPs, respectively.

### Analyses of differentially expressed miRNAs (DEMs) and prediction of target genes

For the aberrantly miRNA expression profile analyses, the web analytical tool, GEO2R, was applied to identify DEMs with fold change (FC) > 2.0 and an adjusted p value <0.01. GEO2R (http://www.ncbi.nlm.nih.gov/geo/geo2r) is an R-based interactive web tool to identify differentially expressed genes via analyzing GEO data [25]. There are several softwares for prediction of miRNA targeting genes, but their algorithms are different and each of them has advantages and disadvantages. Therefore, it is necessary to combine with different software to reduce errors or biases. In this study, miRNA target genes were predicted using TargetScan v7.0 (http://www.targetscan.org/) [26] and miRDB v5.0 (http://www.mirdb.org/miRDB) [27]. Both of them utilize the latest miRNA data provided by miRBase v21. To reduce false-positive results, only common genes predicted by both softwares were chosen as target genes of deregulated miRNA for subsequent analysis.

### Quality control, data preprocessing and analysis of differentially expressed genes (DEGs)

For the analyses of differentially expressed genes, the original data of the series GSE6740 were analyzed using the software Rv3.2.2 (https://www.r-project.org/). Initially, both index, including Relative Log Expression (RLE) and the Normalized Unscaled Standard Error (NUSE), were used to assess the quality of this microarray data [28]. Then, the method of Robust Multi-array Average (RMA) was applied to perform background adjustment, normalization and log transformation of the original microarray data [29]. Finally, the Linear Models for Microarray Data (LIMMA) package (http://bioconductor.org/biocLite.R) was used to identify differentially expressed genes (DEGs), which is a software package for constructing linear regression model [30]. The genes with FC > 1.5 and an adjusted p value <0.05 were regarded as DEGs.

### Functional annotation and pathway enrichment analysis

The dysregulated genes in different disease stages were extracted as DEGs, which needed further functional annotation. Only genes that exhibited significant expression differences (p value <0.05 and FC > 1.5) were functionally annotated. These DEGs were analyzed using Database for Annotation, Visualization, and Integrated Discovery v6.7 (DAVID v6.7) that is a useful bioinformatics enrichment tool for GO terms, KEGG pathway, and gene-disease association (http://david.abcc.ncifcrf.gov/) [31]. To functionally annotate DEGs identified by the aforementioned three comparison groups, Kyoto Encyclopedia of Genes and Genomes (KEGG) pathway and Gene Ontology (GO) were analyzed with DAVID v6.7 [32]. Cytoscape (http://www.cytoscape.org/) was used in miRNA-mRNA network analysis [33].

## Results

### Identification of DEMs for prediction of disease progression during HIV-1 infection

Through a comprehensive analysis of miRNA expression profiling in different disease stages following HIV-1 infection, a list of aberrantly expressed miRNAs was included (Table 1). With at least twofold change and FDR-adjust p value of <0.01, we identified that 7 differentially expressed miRNAs (DEMs) in LTNPs, whose miR-342 was down-regulated and 6 miRNAs (miR-487b, miR-212, miR-494, miR-939, miR-1225 and miR-513a) were overexpressed in the LTNPs, compared with UCs, except of miR-768-5p because it overlaps an annotated snoRNA (HBII-239). Twenty DEMs were identified between CPs and UCs. Twelve miRNAs were higher and 7 DEMs were down-regulated in UCs, compared with CPs, whereas miR-923 that appeared to be a fragment of the 28S rRNA was removed, and miR-768-5p overlapped an annotated snoRNA (HBII-239) was not included. However, only miR-487b was overexpressed in LTNPs when 5 up-regulated miRNAs that also found in the group of CPs versus UCs were excluded. In addition, only miR-630 showed significantly differential expression among LTNPs, UCs and CPs, and the expression level of miR-630 was higher in CPs than that in LTNPs and UCs. It is well known that miR-630 relates to tumor cell growth, proliferation and metastasis [34, 35], involves in growth arrest of cancer cells [36], and can server as a prognostic marker for colorectal cancer [37] and gastric cancer [38], which implies that miR-630 may be a potential biomarker for prediction of disease progression during HIV-1 infection.

**Table 1:** Aberrantly expressed miRNAs and their predicted target gene numbers

Comparison groups	Up-regulated miRNA	Target scan v7.0	miRDB v5.0	Common	Down-regulated miRNA	Target scan v7.0	miRDB v5.0	Common
LTNPs versus UCs	miR-487b-3p	412	26	24	miR-342-5p	3346	238	182
	miR-212-3p	1304	366	134				
	miR-494-3p	5763	504	475				
	miR-939-5p	4170	398	296				
	miR-1225-5p	2412	148	139				
	miR-513a-5p	5509	481	453				
CPs versus UCs	miR-212-3p	1304	366	134	let-7a-5p	354	435	27
	miR-575	3293	238	132	let-7f-5p	101	439	29
	miR-574-5p	3687	246	225	let-7g-5p	120	434	19
	miR-572	679	14	12	miR-342-5p	3346	238	182
	miR-513b-5p	5156	322	306	let-7c-5p	23	435	2
	miR-940	3046	1024	276	let-7d-5p	1062	438	117
	miR-939-5p	4170	398	296				
	miR-638	1866	10	7				
	miR-494-3p	5763	504	475				
	miR-630	3071	182	175				
	miR-513a-5p	5509	481	453				
	miR-1225-5p	2412	148	139				
LTNPs versus CPs	NR	NR	NR	NR	miR-630	3071	182	175

*LTNPs* long-term nonprogressors, *UCs* uninfected controls, *CPs* chronic progressors, *NR* not report

### Analyses of the gene expression profiles of DEMs predicted target genes

Firstly, TargetScan v7.0 and miRDB v5.0 were used to predict deregulated miRNA target genes, and the common genes in both software were chosen. Totally, 1703 common genes were predicted as 7 DEMs target genes in the group of LTNPs versus UCs; 3006 common genes were predicted for 18 DEMs in the group of CPs versus UCs; and 175 target genes in the group of LTNPs versus CPs (Table 1).

**Fig. 1:**
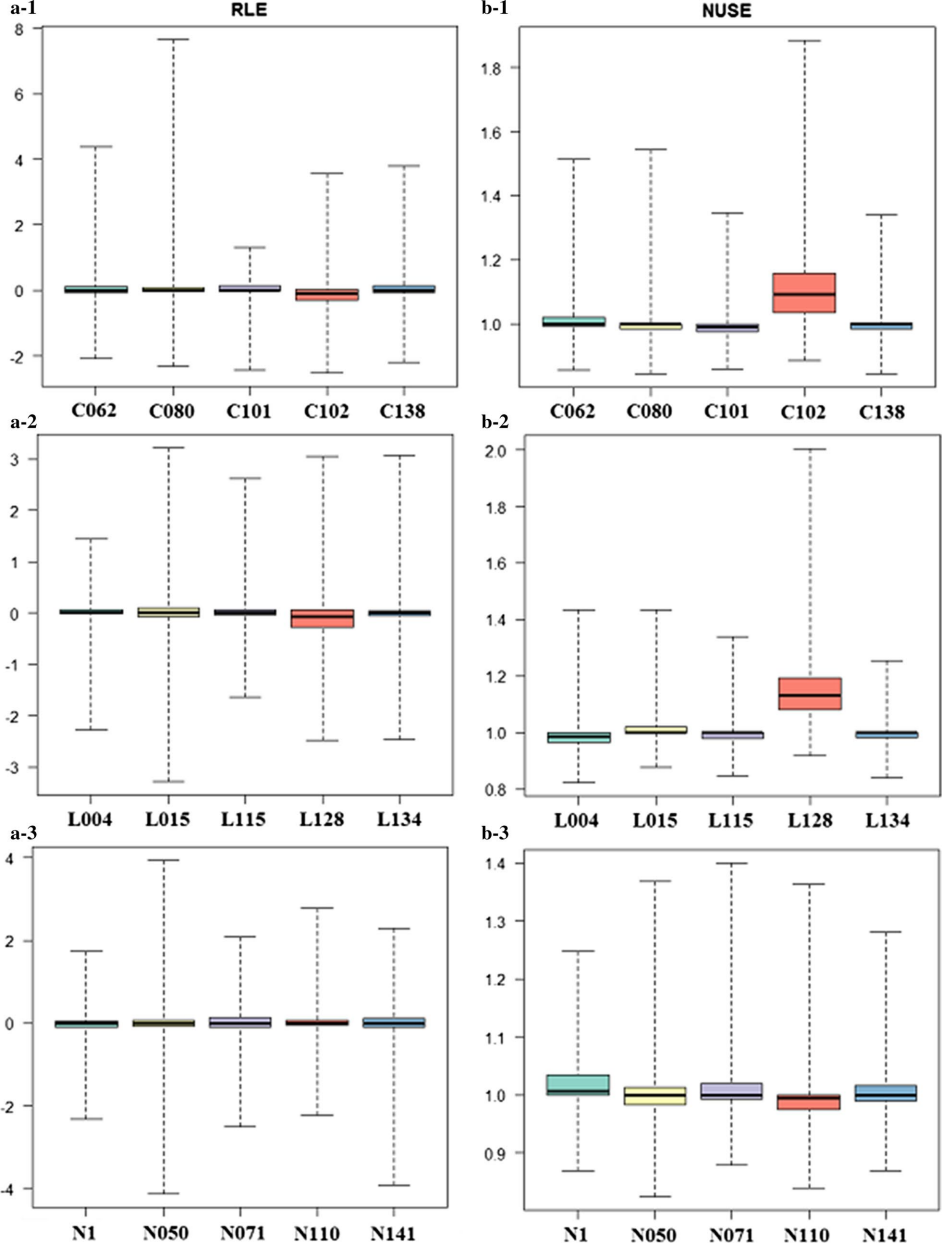
RLE and NUSE box plots of GSE6740. **a** RLE box plots. **b** NUSE box plots. NUSE is a very sensitive measure of noise or variation in the array data. *C* chronic progressors, *L* long-term nonprogressors, *N* uninfected controls

**Fig. 2:**
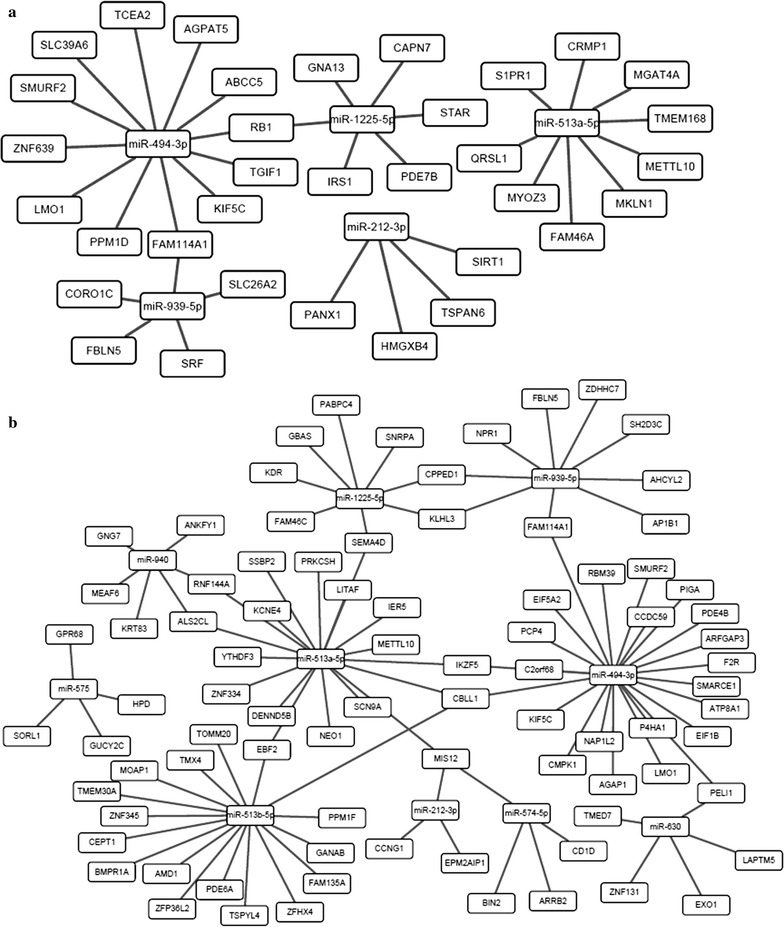
Genetic interactive networks for miRNA/mRNA pair candidates. **a** miRNA-mRNA interaction network from the group of LTNPs versus UCs; **b** miRNA-mRNA interaction network from the group of CPs versus UCs. *CPs* chronic progressors, *LTNPs* long-term nonprogressors, *UCs* uninfected controls

After allowing for overlap between groups, 2629 target genes were predicted from differentially expressed miRNAs, however, the predicted target gene expression profiles still needed to be analyzed in order to elucidate the real miRNA-mRNA relationships in a pairwise manner. Next, we downloaded the series GSE6740 to perform identification of DEGs and functional annotation. To avoid the potential biases caused by inadequate quality of DNA array, both RLE and NUSE box plots were used to check the quality of these DNA arrays. Two DNA arrays GSM155202 (C102, Fig. 1b-1) and GSM155224 (L128, Fig. 1b-2) were excluded by the NUSE box plots analysis because of the arrays quality problems, which were not suitable for subsequent analysis. Finally, the gene expression profiles were divided into three different comparison groups, LTNPs versus UCs, CPs versus UCs, and LTNPs versus CPs, respectively. We identified that 478 genes were differentially expressed in LTNPs and 9 genes (RHOB, NCOA6, ATP8B1, CCL4, SEC31B, PTGER2, AVPR1B, MPI, and LOC285830) were up-regulated in LTNPs, compared with UCs. Besides, 799 differentially expressed genes (DEGs) were identified in the group of CPs versus UCs, and 424 DEGs were found in the comparison group of LTNPs versus CPs. It’s worth noting that 184 unique DEGs were only identified in the group of LTNPs versus CPs, including 38 up-regulated genes in LTNPs, such as CCL22, LILRB3, CCL7/MCP-3, TRAP1, TUBB1 and KLRG1; and 146 down-regulated genes, such as TMPO, BST2, RBX1, CCNA2, OAS2, FOXM1, EZH2, PAFF1, and so on, which may be involved in disease progression during HIV-1 infection (Additional file 2).

### Functional pathway analysis of DEGs in HIV-1 infection

GO and KEGG pathway analyses were performed with DAVID v6.7 to analyzed the differentially expressed genes (Additional file 1), which revealed that the DEGs between LTNPs and UCs were significantly enriched in plasma membrane, cytoplasm and nucleoplasm, including 9 up-regulated genes (RHOB, NCOA6, ATP8B1, CCL4, SEC31B, PTGER2, AVPR1B, MPI, and LOC285830), which involved in plasma membrane part (GO:0044459, p value = 0.016) and plasma membrane (GO:0005886, p value = 0.022). Further, gene ontology biological process (GO BP) analysis indicated that, compared to UCs, DEGs were significantly enriched in CPs’ immune system process (GO:0002376, p value = 1.6 × 10^−8^), defense response (GO:0006952, p value = 6.1 × 10^−5^), response to other organism (GO: 0051707, p value = 3.3 × 10^−13^), response to biotic stimulus (GO: 0009607, p value = 2.7 × 10^−12^), response to virus (GO: 0009615, p value = 9.2 × 10^−9^), response to external stimulus (GO:0006954, p value = 2.6 × 10^−5^), and inflammatory response (GO:0006954, p value = 6.6 × 10^−6^). Additionally, GO BP analysis showed that DEGs between CPs and LTNPs were related to immune system process (GO:0002376, p value = 8.5 × 10^−5^), response to other organism (GO: 0051707, p value = 2.5 × 10^−6^), response to biotic stimulus (GO: 0009607, p value = 9.9 × 10^−6^), response to virus (GO: 0009615, p value = 2.5 × 10^−6^), response to external stimulus (GO:0006954, p value = 4.1 × 10^−4^), and inflammatory response (GO:0006954, p value = 7.1 × 10^−5^), (Additional file 1). These results indicated that, in the CPs group, excessive immune activation may accelerate disease progression in chronic infection (genes: OAS1, ISG15, IFIT1, IFI27, IFI44L, and so on. Additional file 2). Furthermore, the DEGs between different groups were also subjected to KEGG pathway enrichment analysis. The KEGG pathway, RIG-I-like receptor signaling pathway was significantly enriched in CPs, compared to UCs (hsa04622, p value = 0.0038), and LTNPs (hsa04622, p value = 0.0039), revealing excessive innate immune response (genes: AZI2, DDX58, ISG15 and IRF7) in chronic infection compared to that in nonprogression or negative infection (Table 2).

**Table 2:** Enrichment of KEGG pathways with p < 0.05

Comparison groups	Up-regulated genes	Terms	P value	Down-regulated genes	Terms	P value
LTNPs versus UCs	9	NR	NR	469	TGF-beta signaling pathway	0.013
					Complement and coagulation cascades	0.015
					P53 signaling pathway	0.047
CPs versus UCs	97	RIG-I-like receptor signaling pathway	0.0038	702	Ribosome	2.6 × 10^−5^
		O-Glycan biosynthesis	0.0079		Fatty acid elongation in mitochondria	0.0042
		Cytosolic DNA-sensing pathway	0.025		Cytokine-cytokine receptor interaction	0.046
LTNPs versus CPs	118	Beta-Alanine metabolism	0.018	306	Pyrimidine metabolism	0.028
		Cytokine-cytokine receptor interaction	0.035		One carbon pool by folate	0.033
					RIG-I-like receptor signaling pathway	0.039

*KEGG* Kyoto encyclopedia of genes and genomes, *NR* not report

### Screening of inversely correlated miRNA-mRNA pair candidates

Potential target genes identified based on microarray gene expression profiles were included in miRNA-mRNA crosstalk analysis if they met the two following criteria: (1) the expression level of miRNA and target genes are inversely correlated, because miRNAs function to degrade mRNA and/or inhibition of mRNA translation; (2) and the expression of target genes showed at least 1.5-fold change in different comparison groups, and an adjusted p value <0.05. Compared to UCs, we acquired 34 putative down-regulated target genes from up-regulated miRNAs that were identified in LTNPs, and 84 underexpressed genes in CPs (Additional file 2). The functional annotation of putative target genes showed differentially enriched GO terms between LTNPs and CPs. The highly enriched BP terms include regulation of cell communication (GO: 0010646), regulation of signal transduction (GO: 0009966), negative regulation of signal transduction (GO: 0009968), regulation of developmental process (GO: 0050793), and positive regulation of cell differentiation (GO: 0045579) in LTNPs but not UCs, while enzyme linked receptor protein signaling pathway (GO: 0007167), receptor quanylyl cyclase signaling pathway (GO: 0007168), regulation of body fluid level (GO: 0050878), and cellular amino acid derivative metabolic process (GO: 0006575) were enriched in CPs but not UCs. In addition, the most enriched MF terms were ion binding (GO: 0043167), quanylate cyclase activity (GO: 0004383), metal ion binding (GO: 0046872), and cation binding (GO: 0043169) were in CPs, and KEGG pathway analysis found two pathways endocytosis and purine metabolism, indicating miRNA-regulated genes may be involved in metabolism of chronic progressors (Table 3). After combining the gene expression profiles of the miRNA-mRNA pair candidates, the interactive networks of putative miRNA-mRNA pairs constructed with Cytoscape were shown in Fig. 2 and Additional file 3.

**Table 3:** Functional annotation of putative target genes with p < 0.05

Comparison groups		GO ID	Function	P value	KEGG ID	Function	P value
LTNPs versus UCs miR-212-3p, miR-494-3p, miR-939-5p, miR-1225-5p, miR-513a-5p	Biological process	0010646	Regulation of cell communication	0.0042	NR	NR	NR
		0009966	Regulation of signal transduction	0.0079			
		0009968	Negative regulation of signal transduction	0.010			
		0050793	Regulation of developmental process	0.011			
		0045579	Positive regulation of cell differentiation	0.011			
	Cellular component	0044424	Intracellular part	0.046			
	Molecular function	NR	NR	NR			
CPs versus UCs miR-212-3p, miR-575, miR-574-5p, miR-513b-5p, miR-940, miR-939-5p, miR-494-3p, miR-630, miR-513a-5p, miR-1225-5p	Biological process	0007167	Enzyme linked receptor protein signaling pathway	0.024	Hsa04144	Endocytosis	0.025
		0007168	Receptor quanylyl cyclase signaling pathway	0.029	Hsa00230	Purine metabolism	0.046
		0050878	Regulation of body fluid level	0.031			
		0006575	Cellular amino acid derivative metabolic process	0.046			
	Cellular component	0044464	Cell part	0.0058			
		0005623	Cell	0.0058			
		0009898	Internal side of plasma membrane	0.015			
		0044459	Plasma membrane part	0.039			
		0044424	Intracellular part	0.043			
	Molecular function	0043167	Ion binding	0.030			
		0004383	Quanylate cyclase activity	0.034			
		0046872	Metal ion binding	0.040			
		0043169	Cation binding	0.044			
		0046914	Transition metal ion binding	0.049			

*KEGG* Kyoto encyclopedia of genes and genomes, *NR* not report

## Discussion

In our study, we firstly analyzed the differentially miRNAs profiles in LTNPs, CPs and UCs. Based on the cut-off value at >twofold change and the p value at <0.01, we investigated that 6 miRNAs were differentially expressed both in LTNPs and CPs, miR-342-5p (↓), miR-212-3p (↑), miR-494-3p (↑), miR-939-5p (↑), miR-1225-5p (↑), an miR-513a-5p (↑) in LTNPs and CPs, compared with UCs, indicating these deregulated miRNAs may be HIV-1-specific miRNAs of CD4^+^ T cells following HIV-1 infection. We also found that the expression levels of miR-575, miR-574-5p, miR-572, miR-513b-5p, miR-940 and miR-638 were higher in CPs than that in UCs, although they were not altered between LTNPs and CPs. Previous evidence indicated that suppressor of cytokine signaling 1 (SOCS1) protein is a target of miR-572 [39], and Miller et al. [40] have found that the expression level of suppressor of cytokine signaling 1 (SOCS1) protein in CD4^+^ T cells is lower in HIV-1 infected patients than that in healthy people, but SOCS1 mRNA level is higher in HIV-1 infection, indicating miR-572 may be related to sustained immune activation that promoted disease progression and pathogenesis following HIV-1 infection by directly targeting SOCS1. Besides, miR-940 can inhibit the growth of pancreatic ductal adenocarcinoma via targeting MyD88 [41] that involved in IL-33 mediated type 1 helper T cells (Th1) differentiation [42] (Th1 is pivotal in cellular immunity). We confirmed that let-7 family was down-regulated in CPs compared with UCs, which is consistent to findings of Swaminathan et al. [20].

Next, we applied TargetScan v7.0 and miRDB v5.0 to predict target genes of differentially expressed miRNAs and 2629 unique target genes predicted from three different comparison groups. Transcriptomic analysis of ex vivo CD4^+^ T cells from different clinical outcomes during HIV-1 infection, like LTNPs and CPs, we also found higher expression level of interferon-stimulated genes (ISGs), such as ISG-15 [43–45], IFI44, IFI44L, HERC6, IFI6, and so on, in CPs [46], indicating chronic immune activation, which is also differentially expressed between pathogenic (rhesus macaques [47–49]) and non-pathogenic (sooty mangabeys [50] or African green monkeys [51]) SIV infection, demonstrated by highly enriched GO terms and KEGG pathways, including response to virus (GO: 0009615), immune system process (0002376), and RIG-I-like receptor signaling pathway (hsa04622). Our findings confirm earlier studies that showed that a chronic interferon response or immune activation contributed to CD4^+^ T cells loss, pathogenesis and immune exhaustion in HIV-1 chronic infection [43, 44, 52, 53]. Moreover, it has been shown that immune inhibitory molecules, including LAG-3 [54] and CD160 [55], have higher levels in CPs than in LTNPs and UCs and are involved in immune exhaustion that accelerated HIV-1 disease progression. Additionally, we also identified 184 unique DEGs in LTNPs, which were involved in HIV/AIDS disease control or progression, including 38 up-regulated genes such as CCL22 (a soluble HIV-suppressive factor [56], LILRB3 (related to immune protection for HIV-1 infection) [57] and CCL7/MCP-3 (competed for HIV-1 gp120 binding) [58], and 146 down-regulated genes such as TMPO (involved in HIV-1 Tat-induced apoptosis of T cells) [59], BST2 (increased in SIV-infected rhesus monkeys) [60], RBX1 (involved in proteasomal degradation of APOBEC3G) [61], CCNA2 (contributed to loss of SAMHD1 ability to inhibit HIV-1) [62] and some unreported genes such as FOXM1, EZH2 and PAFF1 (Additional file 2).

Further, we analyzed negatively correlated miRNA-mRNA pair candidates, and the potential target genes were selected from the series GSE6740. We identified that thirty-four deregulated target genes with 5 up-regulated miRNAs were identified from the group of LTNPs versus UCs, and eighty-four repressed target genes from 10 up-regulated miRNAs in the group of LTNPs versus UCs, whose expression of miRNA and target genes showed negative correlation. The functional annotation revealed that miRNA-regulated genes may be involved in metabolic processes in chronic infection. There are several studies that have shown that down-regulation of CPPED1 expression improves glucose metabolism in adipocyte [63]; PCP4 plays an anti-apoptotic role in human breast cancer cells [64], and CBLL1 promotes cell proliferation in the early stages of tumor progression [65], whose genes were deregulated in CD4^+^ T cells of HIV-1-infected chronic progressors in our current study. We also demonstrate that the putative miRNA-mRNA pair candidates are involved in disease progression and pathogenesis. Inhibitory cytokine IL-10 contributes to dysregulated cytotoxic T cell function to HIV-1 infection, and IL-10 was verified to be the target gene of let-7 [20], which was down-regulated in CPs, compared with UCs. We have found that dysregulated CD100 in chronic HIV-1 infection, which is the putative target gene of miR-1225a-5p or miR-513a-5p. Loss of Sema4D/CD100 expression plays key roles in dysfunctional immunity during HIV-1 infection [66]. As the positive modulator of cellular apoptosis [67], MOAP1 was down-regulated in chronic infection, which implied that HIV-1 might employ cellular miRNAs to support persistent infection. The ubiquitin ligase Peli1 encoded by PELI1 inversely regulated T lymphocyte activation [68], whose expression level was decreased in our study, partly indicating hyperactivation of CD4^+^ T cells related to pathogenesis in HIV-1 infection [69].

However, we understood that there were limitations in our bioinformatics-based study. There were only 22 subjects (7 LTNPs, 7 CPs and 8 health controls) in the series of GSE24022 for miRNAs analysis and 13 subjects (4 LTNPs, 4CPs and 5 normal controls) in the series GSE6740 for DEGs. It is necessary to recruit more subjects in the future. We also recognized that there were a few differences between two series including the duration of infection, the definitions of disease stages of HIV-1 infection and chronic progression, viral load and CD4^+^ T cell counts. Therefore, it is necessary to be confirmed whether the level of deregulated miRNAs and putative target genes expression is actually altered in distinct disease progression of HIV-1 infection. The bioinformatics-based methods to obtain disease progression-related gene expression profiles and the interactive networks of miRNA-mRNA pair candidates via integrative analysis of miRNA-mRNA expression should be applied in integrative analyses of miRNA-mRNA expression profiles in different stages of HIV-1 infection, which will not only facilitate the understanding of the genetic basis of interaction between HIV-1 and host cells, but lead to the development of genetic markers for prediction of disease progression and therapy of HIV-1 in the future.

## Conclusions

In summary, our integrative bioinformatics study showed that distinct transcriptional profiles in CD4^+^ T cells, including microRNAs and mRNAs, associated with different disease progression during HIV-1 infection, and identified a potential biomarker, miR-630, that may be employed to predict disease progression in HIV-1 infection.

## Additional files

**Additional file 1.** Classification of DEGs according to GO terms with p < 0.05.

**Additional file 2.** Differentially expressed genes identified from the series GSE6740.

**Additional file 3.** Putative target genes of differentially expressed miRNAs identified from the series GSE6740. Different colors of font represent overlapping putative target genes.

## Abbreviations

HIV-1: human immunodeficiency virus 1
AIDS: acquired immunodeficiency syndrome
LTNP: long-term nonprogressor
UC: uninfected control
NP: normal progressor
CP: chronic progressor
ART: antiretroviral therapy
LVL: low viral load
DAVID: database for annotation, visualization and integrated discovery
GEO: gene expression omnibus
miRNA: microRNA
LncRNA: long non-coding RNA
HDF: HIV dependency factors
PCAF: P300/CBP-associated factor
PBMC: peripheral blood mononuclear cell
DEM: differentially expressed miRNA
DEG: differentially expressed gene
GO: gene ontology
BP: biological process
MF: molecular function
CC: cellular component
KEGG: kyoto encyclopedia of genes and genomes
PLE: relative log expression
NUSE: normalized unscaled standard error
RMA: robust multi-array average
LIMMA: linear models for microarray data
FC: fold-change
SOCS1: suppressor of cytokine signaling 1
MyD88: myeloid differentiation factor 88
ISG: interferon-stimulated gene
ISG-15: interferon-stimulated gene 15
IFI44: interferon induced protein 44
IFI44L: interferon induced protein 44 like
HERC6: HECT and RLD domain containing E3 ubiquitin protein ligase family member 6
IFI6: interferon induced protein 6
Th1: type 1 helper T cell
CPPED1: calcineurin like phosphoesterase domain containing 1
PCP4: purkinje cell protein 4
CBLL1: cbl proto-oncogene like 1
Sema4D: semaphoring 4D
MOAP1: modulator of apoptosis 1
