# Correction to: Identification of miRNA-mRNA crosstalk in CD4^+^ T cells during HIV-1 infection by integrating transcriptome analyses

**DOI:** 10.1186/s12967-020-02479-6

**Published:** 2020-08-13

**Authors:** Qibin Liao, Jin Wang, Zenglin Pei, Jianqing Xu, Xiaoyan Zhang

**Affiliations:** Shanghai Public Health Clinical Center & Institutes of Biomedical Sciences, Key Laboratory of Medical Molecular Virology of Ministry of Education/Health, Fudan University, Shanghai, China

## Correction to: J Transl Med (2017) 15:41 10.1186/s12967-017-1130-y

Following publication of the original article [1], the authors identified an error in the affiliation list. The two affiliations should have been merged into one single affiliation:

Shanghai Public Health Clinical Center & Institutes of Biomedical Sciences, Key Laboratory of Medical Molecular Virology of Ministry of Education/Health, Fudan University, Shanghai, China.

The corrected affiliation list is reflected in this Correction.
