# Supplementary material for: Identification of miRNA-mRNA crosstalk in CD4+ T cells during HIV-1 infection by integrating transcriptome analyses
Source: J Transl Med. 2017 Feb 21;15:41. doi: 10.1186/s12967-017-1130-y (PMC5319073; doi:10.1186/s12967-017-1130-y)
Supplement: Supplementary file 1 — Additional file 1. Classification of DEGs according to GO terms with p < 0.05. [file 12967_2017_1130_MOESM1_ESM.docx]

**Table S1. Classification of DEGs according to GO terms with *p*﹤0.05.**

| **Comparison**  **groups** | **Up-regulated genes** | **GO terms** | **GO ID** | **Function** | ***P*-value** | **Down-regulated genes** | **GO terms** | **GO ID** | **Function** | ***P*-value** |
| --- | --- | --- | --- | --- | --- | --- | --- | --- | --- | --- |
| **LTNPs**  **versus**  **UCs** | 9 | Biological  Process | NR | NR | NR | 469 | Biological  Process | 0051726 | Regulation of cell cycle | 0.0067 |
|  |  |  |  |  |  |  |  | 0065009 | Regulation of molecular function | 0.0087 |
|  |  |  |  |  |  |  |  | 0006974 | Response to DNA damage stimulus | 0.0099 |
|  |  |  |  |  |  |  |  | 0015914 | Phospholipid transport | 0.011 |
|  |  |  |  |  |  |  |  | 0050864 | Regulation of B cell activation | 0.011 |
|  |  | Cellular  Component | 0044459 | Plasma membrane part | 0.016 |  | Cellular  Component | 0044424 | Intracellular part | 0.0028 |
|  |  |  | 0005886 | Plasma membrane | 0.022 |  |  | 0005654 | nucleoplasm | 0.0041 |
|  |  |  |  |  |  |  |  | 0005622 | intracellular | 0.0063 |
|  |  |  |  |  |  |  |  | 0005737 | cytoplasm | 0.0092 |
|  |  |  |  |  |  |  |  | 0030018 | Z disc | 0.024 |
|  |  | Molecular  Function | NR | NR | NR |  | Molecular  Function | 0002020 | Protease binding | 0.013 |
|  |  |  |  |  |  |  |  | 0016787 | Hydrolase activity | 0.014 |
|  |  |  |  |  |  |  |  | 0005488 | binding | 0.014 |
|  |  |  |  |  |  |  |  | 0042578 | Phosphoric ester hydrolase activity | 0.015 |
|  |  |  |  |  |  |  |  | 0016805 | Dipeptidase activity | 0.017 |
| **CPs**  **versus**  **UCs** | 97 | Biological  Process | 0051707 | Response to other organism | 3.3×10^-13^ | 702 | Biological  Process | 0009611 | Response to wounding | 2.3×10^-7^ |
|  |  |  | 0009607 | Response to biotic stimulus | 2.7×10^-12^ |  |  | 0006954 | Inflammatory response | 6.6×10^-6^ |
|  |  |  | 0051704 | Multi-organism process | 4.4×10^-9^ |  |  | 0006412 | translation | 2.5×10^-5^ |
|  |  |  | 0009615 | Response to virus | 9.2×10^-9^ |  |  | 0009605 | Response to external stimulus | 2.6×10^-5^ |
|  |  |  | 0002376 | Immune system process | 1.6×10^-8^ |  |  | 0006952 | Defense response | 6.1×10^-5^ |
|  |  | Cellular  Component | 0031225 | Anchored to membrane | 0.0065 |  | Cellular  Component | 0005887 | Integral to plasma membrane | 1.4×10^-5^ |
|  |  |  | 0005737 | cytoplasm | 0.020 |  |  | 0031226 | Intrinsic to plasma membrane | 3.0×10^-5^ |
|  |  |  |  |  |  |  |  | 0044424 | Intracellular part | 1.4×10^-4^ |
|  |  |  |  |  |  |  |  | 0005865 | Striated muscle thin filament | 1.5×10^-4^ |
|  |  |  |  |  |  |  |  | 0022626 | Cytosolic ribosome | 2.0×10^-4^ |
|  |  | Molecular  Function | 0004252 | Serine-type endopeptidase activity | 0.0075 |  | Molecular  Function | 0005515 | Protein binding | 2.1×10^-5^ |
|  |  |  | 0008233 | Peptidase activity | 0.008 |  |  | 0005488 | binding | 8.5×10^-5^ |
|  |  |  | 0046966 | Thyroid hormone receptor binding | 0.011 |  |  | 0003700 | Transcription factor activity | 6.4×10^-4^ |
|  |  |  | 0008236 | Serine-type endopeptidase activity | 0.012 |  |  | 0008135 | Translation factor activity, nucleic acid binding | 0.0014 |
|  |  |  | 0017171 | Serine hydrolase activity | 0.013 |  |  | 0030528 | Transcription regulator activity | 0.0014 |
| **LTNPs**  **versus**  **CPs** | 118 | Biological  Process | 0009611 | Response to wounding | 1.3×10^-5^ | 306 | Biological  Process | 0051707 | Response to other organism | 2.5×10^-6^ |
|  |  |  | 0006950 | Response to stress | 1.3×10^-5^ |  |  | 0009615 | Response to virus | 2.5×10^-6^ |
|  |  |  | 0006954 | Inflammatory response | 7.1×10^-5^ |  |  | 0009607 | Response to biotic stimulus | 9.9×10^-6^ |
|  |  |  | 0009605 | Response to external stimulus | 4.1×10^-4^ |  |  | 0006955 | Immune response | 1.9×10^-5^ |
|  |  |  | 0006898 | Receptor-mediated endocytosis | 4.6×10^-4^ |  |  | 0002376 | Immune system process | 8.5×10^-5^ |
|  |  | Cellular  Component | 0005887 | Integral to plasma membrane | 8.8×10^-4^ |  | Cellular  Component | 0005737 | cytoplasm | 1.2×10^-7^ |
|  |  |  | 0031226 | Intrinsic to plasma membrane | 0.0011 |  |  | 0005622 | intracellular | 1.1×10^-6^ |
|  |  |  | 0044459 | Plasma membrane part | 0.0014 |  |  | 0044424 | Intracellular part | 2.2×10^-6^ |
|  |  |  | 0005737 | cytoplasm | 0.028 |  |  | 0043231 | Intracellular membrane-bounded organelle | 4.7×10^-4^ |
|  |  |  | 0044427 | Chromosomal part | 0.032 |  |  | 0043227 | membrane-bounded organelle |  |
|  |  | Molecular  Function | 0005488 | binding | 0.0049 |  | Molecular  Function | 0003824 | Catalytic activity | 0.0018 |
|  |  |  | 0030246 | Carbohydrate binding | 0.0065 |  |  | 0005488 | binding | 0.0046 |
|  |  |  | 0030246 | Sugar binding | 0.0073 |  |  | 0019783 | Small conjugating protein-specific protease activity | 0.0065 |
|  |  |  | 0005515 | Protein binding | 0.011 |  |  | 0016787 | Hydrolase activity | 0.0085 |
|  |  |  | 0004872 | Receptor activity | 0.017 |  |  | 0003823 | Antigen binding | 0.013 |
