# Supplementary material for: Identification of miRNA-mRNA crosstalk in CD4+ T cells during HIV-1 infection by integrating transcriptome analyses
Source: J Transl Med. 2017 Feb 21;15:41. doi: 10.1186/s12967-017-1130-y (PMC5319073; doi:10.1186/s12967-017-1130-y)
Supplement: Supplementary file 2 — Additional file 2. Differentially expressed genes identified from the series GSE6740. [file 12967_2017_1130_MOESM2_ESM.docx]

**Table S2. Differentially expressed genes identified from the series GSE6740.**

| Comparison group | Up-regulated genes | Down-regulated genes |
| --- | --- | --- |
| LTNPs versus NCs | *RHOB、NCOA6、ATP8B1、CCL4、SEC31B、PTGER2、AVPR1B、MPI、LOC285830* | *HOOK1、KIF5C、ILKAP、ABCG1、CRMP1、PAFAH1B3、PLEKHA4、P2RY4、PDCD5、NUDT1、LRP5L、SLC12A8、C1QA、GRAMD4、SRF、LAMP3、SERPINF2、LRIG2、CHMP2B、CLN5、EPHX1、SFRP5、NFKBIL1、HIST1H4J、GABARAPL1、TTC12、UCK2、ORC1、MKLN1、TLK1、IL2RB、KBTBD2、TPSD1、E4F1、ARPP19、PEPD、SPAG16、SHOX、PLA2G10、PTPLA、EXTL3、LOC92973、SLURP1、CGREF1、HOXA6、SUOX、MRPL15、MBIP、C1orf14、RAB3IL1、APOL6、MTG1、PNMA3、DENND4C、CCR8、RAD51C、BLVRB、TUBB4Q、TMEM168、PRKCE、FBXO46、BCAS2、CSTF1、CCT6B、MSRA、CAPN7、UNKL、RBBP8、SMURF2、LGALS13、MPPED1、TTN、TLR5、RFC5、LRP3、GRAP2、FDX1、FHL2、RB1、PDZD7、SNX4、WNT7A、LOC441601、DNMT1、AMIGO2、FBXO2、PANX1、BAD、SLC5A4、INTS8、C1orf9、ABCB9、CDX4、C6orf35、PTPRU、PRMT3、DAAM2、SLC2A6、PUS1、TIMM13、STAR、MYCN、G6PC2、SLC1A5、DPEP2、SEC61A2、CAMK4、S1PR1、HMGXB4、CDK7、S100A2、SLC25A28、MTERF、CHP、LSG1、FUT8、TAF7L、FLJ38109、CDK2AP2、ZNF330、SUMO4、ZBP1、ARRB1、ANKRD27、YEATS4、LENEP、SLC26A2、TEX13B、TSPAN6、HOMER2、CD79A、SLC7A4、ZNF280A、BRF2、RPS6KB1、ZNF23、NRAS、PLA2G15、EEF1A2、IFITM2、IRS1、DPP6、C21orf96、ABTB2、PRG3、CDKN1A、HEG1、CCDC6、TNFRSF8、ABCC5、PCOLCE、SIRT1、FBLN5、PDE9A、OCM2、HIST1H4E、MYBPC3、ZNF211、STMN1、TICAM1、TTC33、IPP、ZDHHC14、HRASLS2、BCAS3、FIG4、DGKQ、GRAP、ACAT2、CEP192、RAD54L、HTR1A、CNNM2、C3AR1、ZNF432、CACNG2、CYP21A2、HEBP2、ZNF639、HIST1H2AG、SDC3、IGKV3-20、CRYBB2P1、FABP4、NR3C2、DHRS9、PLCH2、MATN4、UNC119B、ATAD5、LMO1、CSTA、SSSCA1、YSK4、DNTT、LPPR3、C5orf13、GPN2、FASTKD5、THAP11、SERINC1、POLD2、VRK2、CP110、NDUFAF4、KIAA0776、TRIM8、TROAP、C22orf26、VAMP3、ZNF143、EXD3、CCNG2、TMEM183A、C1QB、CD5L、PBX3、GIPC1、LOC254896、ALPK1、SPEG、HSPA4、ERICH1、RCAN3、KRT83、PPM1D、FLRT1、SPACA1、TLR2、SEC61G、OXCT2、AMH、ACSIN3、MDFI、GPR162、IFT57、FAM46A、NBR2、BCL6、GMNN、SERPINA6、TMEM159、WDR67、C1orf105、SRSF6、HLA-DOB、CCDC64、PDE7B、PDE4D、UGDH、DNAJC4、SSR3、SOCS2、ICT1、MECR、PVRL2、THAP7、FAM179B、VWF、RFWD3、EIF3J、ZNF671、CA11、CROCC、C2orf47、MYO15B、ZNF10、GPR19、B3GNT2、IGKV4-1、GNA15、DGKG、TMSB15B、OSGEP、KISS1、PHB、PFKFB2、TBCCD1、POLG2、HERC3、CYP2W1、TMEM160、PECR、DNAH17、FBXO24、GTF3C3、EXOC5、NUDT4、AGPAT5、SLC30A1、TCEA2、TEX28、EAF2、GNA13、MAG、GRAMD1B、TCFL5、TET3、NCF1C、MCM9、KLHL1、CDC45、PMM1、MYST2、METTL10、KPNA4、SPINK2、PAIP2B、CDC42EP1、CHAT、RNF19A、FKBP3、MYOZ3、EP300、MATK、C2orf49、CALML3、TMED3、BSN、FKBP5、TRIM5、SLC39A6、RNF25、COL6A2、MRPL13、ASNSD1、PCTP、PRAMEF10、C1QTNF1、TGIF1、TP53BP2、PHYHIP、C17orf53、ARMC6、FAM114A1、ANO10、ID1、TCTN1、ABCA7、TSPYL4、DEPDC6、ACD、CRYAB、P2RY10、MALL、KNTC1、KCNJ9、CASP3、MOCS3、DUSP11、ACADM、NUP155、RDH14、LEFTY1、ADAMTSL4、C22orf30、GMPR、NAPG、SNAP23、SIAH1、SLC7A10、ACVRL1、SLITRK5、BRIX1、C18orf8、RAB33A、C2orf54、PRM1、SEPHS2、LZTFL1、GLA、EXO1、TBC1D16、MARCO、TRIM13、PIN1P1、PDZD8、GDAP1L1、ZNF646、OGFRL1、ZNF696、PDZK1IP1、ZNF350、NAALADL1、CKB、ZNF83、TUBG2、FABP5、OSGEPL1、CFD、MED21、EFNA3、FBXO3、FANCG、KLF11、CARTPT、C9orf82、LASS4、FCGRT、MXRA7、CKAP2、ERCC3、QRSL1、FAM108B1、EXOSC10、LOC100170939、A4GNT、IGFBP6、RND1、TMEM186、COL9A2、TMED7、GRM2、EFCAB6、PGLYRP1、RWDD3、TOX、GADD45G、TCERG1、SCML2、VPS37B、CORO1C、CPEB1、CRYGD、ARFIP1、SENP2、TNNT3、KLKB1、STXBP3、FAM18B1、DROSHA、PCDH17、LOC100506935、BMP10、MICB、MGAT4A、NMD3、TRPC2、USP36、CD226、FLT3、TNFRSF12A、RIOK2、ESPN、GMCL1、CSNK2A2、DBF4、FIP1L1、PSPH、BAG2、LY6G6E、ATP8B2、CNIH4、DOK3、KRT32、MTERFD1、ACOT9、C12orf5、PIBF1、WDR62、RPUSD2、RBPJL、CYP20A1、SMAD7、UMOD、CCR9、CRTAC1、MTMR6、MRPL44* |
| CPs versus NCs | *MYO1E、C14orf159、MRPL52、SEC31B、ZCCHC2、NCF4、C1GALT1C1、MYL6B、AIM2、MED24、USP9Y、LXN、TIPRL、SIRPG、B4GALT5、CCDC47、GMNN、AZI2、DNAJC13、CTSG、TYMP、BYSL、LY6E、PRTN3、SAR1B、KIR2DL2、GALNT7、MTHFD2、ZNF593、KIR3DL3、PTGER2、GIMAP4、CASP7、SPATS2L、NCOA6、TRIB1、LY96、OASL、MX2、MELK、IFI35、TDRD7、MAD2L1、NPDC1、SLPI、UBXN2B、MPO、TIMM22、GLRX2、LOC220594、LAG3、CLDND1、RHOB、FABP5、LGALS3BP、ZWINT、FAM13A、S100P、RRM2、CEACAM6、TRDV3、GPR15、CD24、LTF、IRF7、IFI6、DDX58、POU2AF1、IFIT3、CD160、XAF1、EIF2AK2、AZU1、CX3CR1、IGLL3P、USP18、RNASE2、DDX60、S100A8、IFI44、OAS3、MX1、BPI、HERC5、DEFA4、HERC6、S100A9、OAS1、CEACAM8、ELANE、PLSCR1、ISG15、LAMP3、IFIT1、RSAD2、IFI27、IFI44L* | *SMAD7、KRT1、UNC119B、NELL2、ZNF91、SLC22A2、NR3C2、LY6G6E、FHL2、ZFP36L2、LOC100507630、FMOD、P2RY10、HADHA、IL6R、ZNF184、NEO1、GSTM2、CCL24、CCNJL、UBL3、CRYBB2P1、COL6A2、DEPDC6、ATP8B2、ICT1、KLRB1、ZC4H2、CSNK2A2、MCAT、POLR1E、CRYGD、AOC3、CFD、SERPINA10、DDIT4、GRAP、UXT、CCR6、SORL1、TNFRSF12A、AGAP1、C20orf7、SLC12A8、SLC2A3、COL9A2、BCL11B、ZFP36、LLGL2、GDAP1L1、CALML3、MATK、TUBG2、ABCC4、KRT83、APOBEC3F、C20orf195、BCL6、SLURP1、VWF、LZTFL1、ATRN、IGF2R、WDR62、MICAL1、METTL9、RGN、EIF3L、PHYHIP、CCNH、LMO1、RAB3A、RAB11FIP5、LGALS2、CDK18、RWDD3、PAIP2B、SH2D4A、RFPL3、P4HA1、IRS4、ZFHX4、CYTIP、ITGB2、ID1、JUNB、CASP3、E4F1、NBR2、C13orf15、FIP1L1、TEX28、ZBTB16、OGFRL1、ETV4、ANKRD55、LILRB4、RANBP6、EPHB6、IGBP1、BPGM、IGFBP6、NOTCH4、SLC39A1、SERPINE2、CDKN1A、DMXL1、CLK3、LMNA、RTN3、CARTPT、TBC1D16、SGK1、SLC2A4RG、PIGR、SMEK2、NFIL3、STMN3、PLIN3、SSBP2、TNNT2、C5AR1、PK155、SCML1、LOC728855、SLC28A1、DOK3、RSL24D1、TRIM8、SLITRK5、TET3、LTA4H、GPR45、FBL、PLXDC1、LGTN、PABPC4、TLR2、MEAF6、EPM2AIP1、EHD3、DAP、ATP5A1、DNAH17、DPEP2、KPNA4、RPL4、ZDHHC11、ESPN、RPS8、LOC254896、C14orf93、ZSCAN18、PBX3、CD3G、SIAH1、PADI3、ZNF506、SPACA1、SLC6A11、KLHL3、ZNF768、PPT1、THOC7、MAG、ICAM3、C12orf10、DGKQ、CFP、ZFHX2、ADAMTSL4、PRKCSH、PLEKHO1、QARS、KLKB1、CXCR5、C12orf24、MYO15B、PTPLB、SRSF6、SPINK2、IKZF5、BTG2、MAGIX、GABARAPL1、IER5、SLC7A10、PIPOX、FBXO3、MSRA、FGF4、ABCA5、CBLL1、HAGH、CFB、CBX7、ORC1、NAP1L2、TSPYL1、TUBBP5、AMOTL2、CUX1、ARRB2、TCTN1、LIN7A、PMM1、CLSTN3、PDZD7、BRF1、SH2D3C、XRCC2、DNAJB9、TNP1、UGDH、GAST、SNRPA、TTN、LOC644450、BET1L、TSPYL4、GP6、GBX2、LOC145678、CYTH1、CDX4、HIRA、GNG7、SRY、KCNQ1、FBXW7、XRCC1、TEAD3、RPL23AP32、TPM2、CCDC59、MTERF、RCAN3、PPA1、TGFBR2、PRM1、BCL2A1、WNT7A、C8orf44、MDFI、CYP2R1、ELK3、AMIGO2、PLEKHA1、RPS9、NPAS1、FAM114A1、FABP4、ACN9、NPR1、LPPR3、USP5、DDOST、KCNF1、RBL2、STMN1、C1orf114、ZNF248、ATP13A2、LY86、MIS12、LCE2B、ANO10、AREG、ACTR1B、RPL15、TICAM1、F2R、KDM1A、ABLIM1、EIF3F、OTUD7B、C19orf53、CREBBP、PRR7、FLI1、RPL35、EMR1、ATP8A1、HOXC13、DVL1、MZT2A、PTP4A1、PEPD、EIF3D、SPTBN4、RPL5、TENC1、NCF1C、KDR、BCL7B、BRP44、KIAA1644、LHX1、RASSF2、ACVRL1、CKAP4、ZDHHC14、OLA1、FBLN5、FBRS、LY6D、HCG4、EIF3H、RND1、KCTD12、RPL23AP7、DNAJC4、FBXO24、UNC13A、MEIS3P1、GPAA1、KLF9、LOC100093698、XBP1、SCN9A、GPER、CAMK4、LENEP、HIST1H3C、KCNIP2、DGKD、CRY1、KLHDC2、ZNF385D、AP1B1、GSTM1、IL5、MAN2B2、MAN2B1、C11orf63、TOPORS、CSN1S1、SCPEP1、PLXNB3、OMP、APOC3、CFHR4、RPL8、HABP4、TAF7、NSA2、CLEC7A、BAG2、PAFAH2、CCNI、PHB2、MAST4、RPS21、KHDRBS3、PPM1F、DDX42、PPFIA4、SESN1、IFNB1、GPR64、CABP2、ADRA2C、DDX25、TM2D3、KISS1、RPL10A、MTCP1NB、TOB1、SLC2A6、DUX1、SSTR1、TMED7、PFDN5、EIF5A2、SLC25A6、ERGIC3、RPL10L、OR10H1、MYLPF、RGS19、RBM17、TTC19、GFER、SEMA4D、SMARCE1、EIF4B、TCFL5、SLC16A10、OR2H2、FLT3、AP1S2、C11orf2、RAE1、ZNF345、EIF3K、SLC30A1、SENP7、PDPK1、TULP1、LRRC47、ALS2CL、TNNI3、SAE1、LAMB2、DHRS3、PIK3IP1、IPO5、YBX1、ADAM5P、MMP28、LILRA6、CD5、TOMM20、CCR7、PRCP、FKSG2、PHF17、UTF1、FAH、NUBPL、PRAMEF10、TYRO3、NACA2、ZNF415、MIR622、C1orf14、MAP3K3、GUCY2C、PIN1P1、C14orf132、MGLL、BRIX1、C2orf68、HSD17B11、C8orf33、THAP7、MAGEA4、ZNF302、C1orf9、GPR68、RBM15B、ZC3HAV1、TSPAN8、SLC17A4、EIF1B、BMPR1A、ZHX2、ZNF550、MSLN、THRAP3、LEPREL2、AHCYL2、SLC7A5、IL1RAP、SOCS3、RNF144A、PDE6A、AKR1C3、SLC1A3、BTBD3、NCOR2、HBEGF、ZNF135、PLP2、PRB3、SMURF2、DNAJC28、IMPDH2、ZNF23、KIF5C、KCNE4、SEC14L2、AIM1L、PTPRM、RBM39、HIST1H2AJ、FAM46C、CDKN2AIP、LIFR、ZNF250、PYGM、TEC、GRIK1、SMU1、ZNF471、RPA1、TTC30A、SEMA3B、PCDH17、SRSF5、LAPTM5、EREG、SEMA4C、C8orf55、LAMP1、TNFRSF1B、RPL6、ZNF426、ANPEP、PTPRO、AZGP1P1、B3GNT2、ITFG2、FAU、ATF1、ITGB1BP3、ASAP3、ZFYVE21、PCYOX1、LOC80054、CPPED1、SLC25A28、SKIV2L、ORC6、ANKFY1、LYPD1、CDHR2、TAZ、ITPA、RPGR、RORA、ARMC6、PELI1、PDE4B、HPD、ZNF334、MYL3、GBAS、GCKR、ATXN2L、WWC2、FKBP6、RPS7、CD3E、KRT8、MEPE、ACR、PER1、NAAA、ARMC7、TNFRSF1A、TAF1B、CLEC4A、C9orf144、EEF1B2、BANP、ATP9A、RPS5、C11orf21、SLC25A5、PITPNC1、GSTP1、TRPV6、LITAF、SH3BGR、RPS14P3、METTL10、DUSP11、ITIH3、TNNC1、ZNF131、TSC22D3、EMP1、DUS1L、UXS1、OR3A2、NLE1、CEPT1、APP、PLEKHF2、MAST3、HSPB1、CDKN1B、UBXN1、ALCAM、ENTPD2、TPM3、TMEM30A、ZNF322B、IFITM2、CLASRP、ERC2、PPP4R2、GANAB、RECQL4、CMPK1、DUSP13、FAM75C2、GAB2、ASTE1、CEBPD、ICK、ERF、AMD1、HAND1、CHAF1B、RBBP6、MOAP1、MAP2K1、GNL3、ZSCAN16、ROBO3、DTX2、ZNF643、ANKHD1、YTHDF3、TMEM144、CYP4F11、ITGAE、C14orf139、CXCR2、CCT4、UMOD、NGF、LIPA、ARFGAP3、UBE2E3、TRIM28、ONECUT2、R3HCC1、MOCS1、AGER、INSRR、ACAA2、DENND5B、C3orf64、INA、PARP6、FAM120C、BANK1、RFXAP、CD48、IPP、EGR3、HEBP2、PMPCA、C14orf104、LECT1、SOX10、LEPROTL1、HCK、PTCRA、EBF2、USP36、ZNF238、RUNDC3B、BIN2、ATP1B4、C19orf40、BTD、USP47、CYSLTR1、USE1、RRAGB、KRT8P12、MAGEH1、PRX、SIGLEC5、FOXO4、CD1D、PCSK5、RIBC2、EXO1、CEP68、RABAC1、CCNG1、ABO、POU3F3、MLLT1、ZYX、GTF2F2、MXI1、ZDHHC7、UBXN8、SNAI1、PCP4、ZBTB48、OXTR、ZNF259P1、EDF1、MATN4、SLC13A4、LOC202181、HADHB、TRABD、C1QTNF1、RDH8、FZD3、SCARA3、PIGA、IQCC、FAM135A、C5orf54、C2、ZNF76、MALT1、KRT10、FAIM2、TMX4、ST20、PJA1、IFNW1* |
| LTNPs versus CPs | *TUBB1、SLC22A2、PTPRM、ZNF91、LOC285830、SLC28A1、ZFP36L2、IL6R、FCER1A、KRT1、JUNB、SORL1、LMNA、RPL23AP7、DDIT4、SGK1、PK155、PABPC4、AGAP1、ICAM3、CENPB、HAGH、TNNT2、DPYD、SERPINA10、NEDD4、ATXN2L、HADHA、GALNT6、RBL2、KIAA1598、RTN3、EDEM1、RDH16、SIGLEC5、CD3E、CDK18、INF2、RPL4、LOC729164、RAB3A、CCR7、NEO1、C20orf195、KLRG1、UNC119B、HAMP、UXT、LOC728855、ARRB2、CCNJL、SMEK2、KIR2DL5A、CD5、IGF2R、GSTM1、GP6、CLSTN3、ADRB2、LTA4H、LOC100507397、MAP3K3、IARS2、SULT1E1、GSTM2、MYO9A、PPIC、KCNE2、LGTN、LY75、EPM2AIP1、FGF4、RFPL3、FANCC、FBL、CBX7、TRAP1、QARS、RALYL、EIF3L、CSGALNACT1、FHL2、CCNI、HSD17B11、LILRB3、DDX25、APBB1IP、CCL22、MMRN1、C14orf139、FMOD、MYLPF、STMN3、TM2D3、PLEKHA1、EIF3K、DHRS3、CKAP4、ESYT1、ZC3HAV1、FBXL7、LOC202181、CCL7、BCL11B、EVX1、ZC4H2、AOC3、RNPEPL1、LOC100287590、DAP、C20orf7、ABLIM1、OAT、LIFR、CCL24、TNFRSF1B、RPA1、CCND1* | *IFIH1、POLA2、NOC3L、ACE、TRPM6、OSBPL11、TMPO、IFIT5、ABCG1、CD79A、SNRNP25、LASS4、EIF4EBP1、MRPL34、ONECUT1、NMI、FOXM1、SHFM1、C3orf14、DAAM2、FAM131A、STAP2、FAM108B1、ADORA1、CD5L、RNF185、SC4MOL、RCOR3、GRM2、CHP、UTP11L、SPP2、ZNF143、KNTC1、IDH1、CYP19A1、BARD1、UBE2C、UGT2B28、C14orf101、MNDA、ERCC3、WDR19、C21orf91、KIF17、LUZP2、NUP37、C13orf34、FAR2、SP100、MMP17、EIF1AY、TEX13B、ZNF264、CLDN10、UTP6、RALA、C11orf75、USP8、PRTN3、TRIP4、HMMR、CCDC28A、RBX1、MECR、DCPS、RNF19A、RBPJL、BYSL、OR7E47P、CTNNB1、JRKL、SEC61A2、PEX11B、TRIM13、GJC1、PLEKHA2、NUDT1、ANKMY2、HSPE1、TRAPPC4、STAP1、PGLYRP1、ZNF330、CPOX、CENPM、PTRH2、CRTAC1、CRYAB、C2orf43、CCDC47、TRPC2、CD1A、MGAT4A、CRYZ、CCNB2、ASNSD1、KLRD1、GAPDHS、BSN、MRPL35、DBF4、FAM46A、POLG2、GTF2H5、FEN1、RAD51C、ACTL7A、RIOK2、PSMA6、NINJ2、SCO2、PAAF1、C17orf53、ZNF280A、CTNNA2、KIN、GTPBP2、MINA、ARMC1、NFS1、HPS5、DHRS9、C1orf105、C6orf123、PER3、TRDMT1、TYMS、TMED3、BST2、DENND4C、MAD2L1BP、MTHFD1、TIMM23、AZI2、TNNT3、CCNA2、C12orf5、DEF8、ZNF510、PELI2、C22orf24、PARP12、AGPS、USP9Y、IER3IP1、TRIM38、FUT8、MARK3、SNRPD1、RTP4、C1GALT1C1、TK1、CHMP5、MTERFD1、ABTB2、KCNJ9、SLC39A6、GPR19、BEX1、BTN2A3、NCF4、SENP2、AIM2、EZH2、LONRF3、SUMO4、KCNS1、C21orf96、DHX29、RFWD3、KIAA0664L3、OAS2、SAMD9、FKBP3、CAV1、NMD3、MOCS3、SOCS2、NDUFAF4、ALAS1、HIST1H3I、RARS、VRK2、MYL6B、YEATS2、NRAS、MXRA7、DFFB、LTN1、APIP、SSSCA1、G6PC2、MAD2L1、ARG1、GMCL1、SPIB、ZNF593、C2orf54、TYMP、C18orf8、NUSAP1、ZNF273、C22orf30、TPX2、TLK1、EXOSC10、GAR1、IFI35、LY6E、CNIH4、FCHSD2、TIMM13、RBBP8、KLHL2、MTHFD2、TSPYL5、ZCCHC2、MELK、SLC26A2、GLA、ATAD2B、IGKC、LGALS3BP、OASL、CASP7、TIPRL、SEPHS2、IRF7、MTMR6、SPATS2L、KLRK1、MRPL44、DDX58、THUMPD2、GALNT7、KIR3DL3、UBXN2B、CYP20A1、TRIB1、SLPI、LOC100170939、KIAA0753、GRAMD1B、LAG3、KIAA0101、STXBP3、LAP3、ARFIP1、LTF、S100P、LXN、MX2、IGKV4-1、C12orf11、CX3CR1、LY96、ZBP1、CD24、SAR1B、ACOT9、LOC220594、KIR2DL2、ZWINT、IGL@、DROSHA、CEACAM6、ACADM、CPEB1、RDH14、IGLV2-23、GMNN、TDRD7、GLRX2、HERC6、MS4A3、CCR9、RRM2、POU2AF1、RNASE2、IFIT3、CD160、IFI6、TRDV3、GPR15、AZU1、MX1、EIF2AK2、FABP5、DDX60、OAS3、USP18、XAF1、IGLL3P、ISG15、IFI44、BPI、HERC5、OAS1、IGJ、PLSCR1、LAMP3、RSAD2、IFIT1、IFI27、IFI44L* |
