# Supplementary material for: Identification of miRNA-mRNA crosstalk in CD4+ T cells during HIV-1 infection by integrating transcriptome analyses
Source: J Transl Med. 2017 Feb 21;15:41. doi: 10.1186/s12967-017-1130-y (PMC5319073; doi:10.1186/s12967-017-1130-y)
Supplement: Supplementary file 3 — Additional file 3. Putative target genes of differentially expressed miRNAs identified from the series GSE6740. Different colors of font represent overlapping putative target genes. [file 12967_2017_1130_MOESM3_ESM.docx]

**Table S3. Putative target genes of up-regulated miRNAs identified from the series GSE6740.**

| **Comparison**  **groups** | **Up-regulated**  **miRNA** | **GSE6740** | **Log_2_FC** | **Adjust**  ***p*-value** | **GSE6740** | **Log_2_FC** | **Adjust**  ***p*-value** |
| --- | --- | --- | --- | --- | --- | --- | --- |
| **LTNPs vs. UCs** | miR-487b-3p | - | - | - | - | - | - |
|  | miR-212-3p | *PANX1* | -0.61 | 1.67×10^-4^ | *SIRT1* | -0.64 | 1.97×10^-2^ |
|  |  | *TSPAN6* | -0.63 | 1.43×10^-4^ | *HMGXB4* | -0.62 | 5.53×10^-3^ |
|  | miR-494-3p | *ABCC5* | -0.64 | 5.01×10^-3^ | *FAM114A1* | -0.76 | 6.17×10^-3^ |
|  |  | *ZNF639* | -0.66 | 2.00×10^-5^ | *LMO1* | -0.66 | 5.59×10^-5^ |
|  |  | *RB1* | -0.61 | 1.72×10^-3^ | *KIF5C* | -0.59 | 2.62×10^-3^ |
|  |  | *TCEA2* | -0.73 | 2.53×10^-3^ | *PPM1D* | -0.68 | 1.00×10^-2^ |
|  |  | *TGIF1* | -0.75 | 1.00×10^-2^ | *SMURF2* | -0.61 | 1.71×10^-2^ |
|  |  | *AGPAT5* | -0.73 | 3.51×10^-3^ | *SLC39A6* | -0.75 | 6.98×10^-4^ |
|  | miR-939-5p | *SLC26A2* | -0.63 | 1.08×10^-4^ | *SRF* | -0.59 | 1.04×10^-4^ |
|  |  | *CORO1C* | -0.87 | 1.84×10^-2^ | *FBLN5* | -0.64 | 2.98×10^-2^ |
|  |  | *FAM114A1* | -0.76 | 6.17×10^-3^ |  |  |  |
|  | miR-1225-5p | *IRS1* | -0.64 | 7.60×10^-5^ | *CAPN7* | -0.61 | 2.42×10^-2^ |
|  |  | *STAR* | -0.62 | 2.88×10^-5^ | *GNA13* | -0.73 | 2.78×10^-2^ |
|  |  | *RB1* | -0.61 | 1.72×10^-3^ | *PDE7B* | -0.70 | 7.67×10^-5^ |
|  | miR-513a-5p | *S1PR1* | -0.62 | 1.13×10^-2^ | *METTL10* | -0.73 | 9.33×10^-4^ |
|  |  | *FAM46A* | -0.69 | 1.91×10^-3^ | *MKLN1* | -0.59 | 1.09×10^-2^ |
|  |  | *MGAT4A* | -0.92 | 1.83×10^-3^ | *QRSL1* | -0.82 | 9.99×10^-4^ |
|  |  | *CRMP1* | -0.59 | 2.35×10^-4^ | *TMEM168* | -0.60 | 5.27×10^-4^ |
|  |  | *MYOZ3* | -0.74 | 3.16×10^-5^ |  |  |  |
| **CPs vs. UCs** | miR-212-3p | *CCNG1* | -0.59 | 4.65×10^-3^ | *EPM2AIP1* | -0.81 | 3.19×10^-3^ |
|  |  | *MIS12* | -0.72 | 8.21×10^-3^ | - | - | - |
|  | miR-575 | *HPD* | -0.62 | 1.71×10^-4^ | *GUCY2C* | -0.66 | 7.27×10^-5^ |
|  |  | *SORL1* | -0.98 | 1.47×10^-3^ | *GPR68* | -0.65 | 2.08×10^-3^ |
|  | miR-574-5p | *ARRB2* | -0.76 | 4.93×10^-3^ | *BIN2* | -0.60 | 7.95×10^-3^ |
|  |  | *CD1D* | -0.59 | 2.20×10^-3^ | *MIS12* | -0.72 | 8.21×10^-3^ |
|  | miR-513b-5p | *PDE6A* | -0.64 | 1.04×10^-3^ | *ZFP36L2* | -1.19 | 2.74×10^-3^ |
|  |  | *TOMM20* | -0.66 | 2.54×10^-3^ | *ZFHX4* | -0.88 | 1.42×10^-4^ |
|  |  | *TMX4* | -0.59 | 2.39×10^-3^ | *GANAB* | -0.61 | 1.88×10^-2^ |
|  |  | *BMPR1A* | -0.64 | 2.53×10^-4^ | *TMEM30A* | -0.61 | 8.78×10^-3^ |
|  |  | *ZNF345* | -0.67 | 1.22×10^-4^ | *PPM1F* | -0.68 | 2.14×10^-4^ |
|  |  | *CBLL1* | -0.77 | 2.05×10^-2^ | *MOAP1* | -0.60 | 5.07×10^-3^ |
|  |  | *TSPYL4* | -0.75 | 1.15×10^-3^ | *CEPT1* | -0.61 | 7.49×10^-4^ |
|  |  | *EBF2* | -0.60 | 4.1×10^-4^ | *AMD1* | -0.61 | 5.17×10^-3^ |
|  |  | *FAM135A* | -0.59 | 6.37×10^-5^ |  |  |  |
|  | miR-940 | *ANKFY1* | -0.62 | 6.37×10^-5^ | *MEAF6* | -0.81 | 1.58×10^-4^ |
|  |  | *RNF144A* | -0.64 | 1.30×10^-3^ | *ALS2CL* | -0.66 | 3.32×10^-3^ |
|  |  | *GNG7* | -0.74 | 7.91×10^-5^ | *KRT83* | -0.95 | 4.06×10^-4^ |
|  | miR-939-5p | *ZDHHC7* | -0.59 | 8.37×10^-4^ | *FAM114A1* | -0.73 | 9.08×10^-3^ |
|  |  | *AP1B1* | -0.69 | 5.26×10^-4^ | *SH2D3C* | -0.75 | 8.40×10^-5^ |
|  |  | *NPR1* | -0.73 | 1.74×10^-3^ | *CPPED1* | -0.62 | 7.44×10^-5^ |
|  |  | *AHCYL2* | -0.64 | 6.74×10^-5^ | *KLHL3* | -0.79 | 1.46×10^-3^ |
|  |  | *FBLN5* | -0.70 | 1.22×10^-2^ |  |  |  |
|  | miR-494-3p | *CCDC59* | -0.74 | 1.68×10^-3^ | *F2R* | -0.72 | 3.49×10^-2^ |
|  |  | *NAP1L2* | -0.76 | 2.78×10^-2^ | *PDE4B* | -0.62 | 7.18×10^-3^ |
|  |  | *IKZF5* | -0.78 | 1.55×10^-2^ | *ARFGAP3* | -0.60 | 5.03×10^-5^ |
|  |  | *CBLL1* | -0.77 | 2.05×10^-2^ | *P4HA1* | -0.88 | 1.85×10^-4^ |
|  |  | *EIF5A2* | -0.67 | 7.20×10^-5^ | *C2orf68* | -0.65 | 3.52×10^-4^ |
|  |  | *PELI1* | -0.62 | 3.55×10^-2^ | *PCP4* | -0.59 | 6.17×10^-3^ |
|  |  | *KIF5C* | -0.64 | 5.42×10^-3^ | *FAM114A1* | -0.73 | 9.08×10^-3^ |
|  |  | *SMURF2* | -0.64 | 3.56×10^-2^ | *RBM39* | -0.64 | 3.41×10^-3^ |
|  |  | *EIF1B* | -0.64 | 1.08×10^-3^ | *AGAP1* | -0.97 | 8.13×10^-5^ |
|  |  | *PIGA* | -0.59 | 2.37×10^-3^ | *LMO1* | -0.89 | 6.17×10^-5^ |
|  |  | *CMPK1* | -0.61 | 8.20×10^-4^ | *SMARCE1* | -0.67 | 1.33×10^-3^ |
|  |  | *ATP8A1* | -0.71 | 1.73×10^-4^ |  |  |  |
|  | miR-630 | *ZNF131* | -0.61 | 2.14×10^-2^ | *EXO1* | -0.59 | 2.31×10^-4^ |
|  |  | *TMED7* | -0.67 | 9.26×10^-3^ | *PELI1* | -0.62 | 3.55×10^-2^ |
|  |  | *LAPTM5* | -0.63 | 3.43×10^-3^ |  |  |  |
|  | miR-513a-5p | *SEMA4D* | -0.67 | 6.32×10^-3^ | *SSBP2* | -0.83 | 3.76×10^-3^ |
|  |  | *IKZF5* | -0.78 | 1.55×10^-2^ | *PRKCSH* | -0.79 | 1.63×10^-3^ |
|  |  | *YTHDF3* | -0.60 | 4.55×10^-2^ | *NEO1* | -1.09 | 2.08×10^-4^ |
|  |  | *CBLL1* | -0.77 | 2.05×10^-2^ | *EBF2* | -0.60 | 4.1×10^-4^ |
|  |  | *ZNF334* | -0.62 | 1.04×10^-4^ | *IER5* | -0.78 | 1.15×10^-2^ |
|  |  | *RNF144A* | -0.64 | 1.30×10^-3^ | *ALS2CL* | -0.66 | 3.32×10^-3^ |
|  |  | *LITAF* | -0.61 | 4.30×10^-3^ | *MIS12* | -0.72 | 8.21×10^-3^ |
|  |  | *METTL10* | -0.61 | 1.26×10^-3^ | *KCNE4* | -0.64 | 6.17×10^-5^ |
|  |  | *DENND5B* | -0.60 | 6.37×10^-5^ | *SCN9A* | -0.69 | 6.37×10^-5^ |
|  | miR-1225-5p | *KDR* | -0.71 | 6.37×10^-5^ | *GBAS* | -0.62 | 5.13×10^-4^ |
|  |  | *SEMA4D* | -0.67 | 6.32×10^-3^ | *FAM46C* | -0.64 | 3.34×10^-2^ |
|  |  | *PABPC4* | -0.82 | 1.78×10^-3^ | *CPPED1* | -0.62 | 7.44×10^-5^ |
|  |  | *SNRPA* | -0.75 | 6.38×10^-4^ | *KLHL3* | -0.79 | 1.46×10^-3^ |

Different colors of font represent different overlapping putative target genes.
